# Gestational Diabetes and Risk Assessment of Adverse Perinatal Outcomes and Newborns Early Motoric Development

**DOI:** 10.3390/medicina57080741

**Published:** 2021-07-22

**Authors:** Milan Lackovic, Biljana Milicic, Sladjana Mihajlovic, Dejan Filimonovic, Aleksandar Jurisic, Ivana Filipovic, Marija Rovcanin, Maja Prodanovic, Dejan Nikolic

**Affiliations:** 1 Clinical Hospital Center “Dr. Dragiša Mišović”, 11000 Belgrade, Serbia; milan.lackovic@dragisamisovic.bg.ac.rs (M.L.); sladjana.mihajlovic@dragisamisovic.bg.ac.rs (S.M.); ivana.filipovic@dragisamisovic.bg.ac.rs (I.F.); 2 Faculty of Medicine, University of Belgrade, 11000 Belgrade, Serbia; ginekologija.misovic@gmail.com (D.F.); mihajlovicobg@gmail.com (A.J.); rovcanin.marija@gakfront.org (M.R.); 3 Faculty of Dental Medicine, University of Belgrade, 11000 Belgrade, Serbia; biljana.milicic@sbb.rs; 4 Obstetrics/Gynecology Clinic “Narodni Front”, 11000 Belgrade, Serbia; 5 Cardiology Clinic, Emergency Center, University Clinical Center of Serbia, 11000 Belgrade, Serbia; dejan.p.nikolic@med.bg.ac.rs; 6 Physical Medicine and Rehabilitation Department, University Children’s Hospital, Tirsova 10, 11000 Belgrade, Serbia

**Keywords:** gestational diabetes mellitus, pregnancy, perinatal outcomes, early motoric development, infants

## Abstract

*Background and Objectives*: The aim of this study was to analyze the presence of gestational diabetes mellitus (GDM) on maternal and fetal perinatal parameters, as well to evaluate the influence of GDM on neonatal early motoric development. *Materials and Methods*: In this prospective study, we evaluated 203 eligible participants that were admitted to obstetrics department for a labor. GDM was assessed by evaluation of maternal parameters, fetal parameters, as well its impact on infants early motoric development (Alberta Infant Motor Scale—AIMS). *Results*: Presence of GDM was significantly positively associated with: pre-pregnancy weight, obesity degree, weight at delivery, gestational weight gain (GWG), body mass index (BMI) at delivery, GWG and increased pre-pregnancy BMI, glucose levels in mother’s venous blood after the delivery, positive family history for cardiovascular disease, pregnancy-related hypertension, congenital thrombophilia, drug use in pregnancy, large for gestational age (LGA), mode of delivery (Cesarean section and instrumental delivery). Likewise, GDM association was detected for tested ultrasound parameters (biparietal diameter (BPD), head circumference (HC), abdominal circumference (AC), femoral length (FL)), length at birth, birth weight, newborn’s head circumference, newborn’s chest circumference, AIMS supination and pronation at three months, AIMS supination, pronation, sitting and standing at six months. Only Amniotic Fluid Index and AIMS supination at three months of infant’s age remained significantly associated in multivariate regression model. *Conclusions*: The presence of significant positive association of numerous tested parameters in our study on perinatal outcomes and early motoric development, points to the necessity of establishing appropriate clinical decision-making strategies for all pregnant woman at risk and emphasize the importance of providing adequate glycaemia control options and further regular follow ups during the pregnancy.

## 1. Introduction

Gestational diabetes mellitus (GDM) is considered to be among most frequent metabolic disorders in pregnancy and it can lead to complications related to the health of the mother and offspring [1]. Gestational diabetes is defined as carbohydrate intolerance that results in hyperglycemia of variable severity that occurs during the pregnancy [1]. GDM is multifactorial disease with the complex influence of both gene and environmental factors. Some of these factors include overweight/obesity, advanced age of pregnant women, a positive family history of insulin resistance and/or diabetes and new “modern” lifestyle [2,3]. Identifying risk factors may represent an opportunity for a prompt prevention and early intervention.

Despite recently proposed new screening strategies and biomarkers for GDM detection, there is still a lack of universal, uniform approach in the screening, diagnosis, and monitoring of GDM. Newly published studies give us controversies regarding appropriate tests, cut off values and screening timing. Overall oral glucose tolerance test (OGTT) is the most widely used biomarker for GDM diagnosis, whereas monitoring of the glycated hemoglobin (HbA1c) levels is proposed as the most appropriate biomarker for GDM course monitoring [4,5,6,7,8]. Disease management is most commonly provided by a diet-hygiene regime, diet, and exercise. If the glucose level could not not be controlled with lifestyle interventions, it is proposed to introduce oral antidiabetic drugs (metformin or glyburide) or insulin. American Diabetes Association (ADA) [9] and the American College of Obstetricians and Gynecologists (ACOG) [10] preferably recommend initiation of pharmacotherapy with insulin, but there are conditions when implementation of insulin is impractical or not possible. In these occasions, metformin and glyburide use is recommended [11,12,13,14,15,16,17,18], since they have demonstrated short-term safety during pregnancy. With the increasing prevalence of GDM worldwide and a wide range of adverse maternal, fetal, and neonatal outcomes [19], it is necessary to establish accurate and comprehensive recommendations for prevention, diagnosis, monitoring, and treatment of GDM.

The presence of GDM is considered to be the potential risk factor for less favorable outcomes of pregnancy as well as infant development, the aim of this study was to evaluate the impact of GDM on maternal, fetal, neonatal, and infant parameters. Additionally, we aimed to evaluate the influence of GDM presence on an infant early motor development.

## 2. Methods

### 2.1. Study Group

This study enrolled 203 eligible participants—“mother-infant” pairs who were followed up at the University hospital “Dr. Dragisa Misovic” in Belgrade from 1 August 2019 to 31 March 2020. “Mother-infant” pairs were randomly selected from the computer data base. Every fifth “mother-infant” pair was included. The study was implemented in accordance with the International Code of Medical Ethics of the World Medical Association (Declaration of Helsinki). Prior the inclusion in the study, the nature and objectives of the study protocols were explained and were fully understood by willingly obtained informed consent by mothers. Women could leave the study at any time. This research obtain approval of the Institutional Review Board (IRB) (No. 01-14706/19, Date: 22 November 2019). The study started in august 2019 where pregnant women were routinely checked-up and screened according to the standard hospital procedures that needed to be done prior to labor and after delivery. The request for the IRB approval was applied prior to the initial evaluation of infants that were three months of age after delivery for motoric development testing, since this exam was not part of the routine screening, check-up, and evaluation after delivery at the hospital. After the IRB approval, the informed consent was obtained, and the patient’s medical history was evaluated and gathered with the infant’s data of motoric development and included in study analysis.

We randomly selected participants from the data base of the hospital’s obstetric ward, where each fifth participant admitted for delivery was included.

Inclusion criteria were: pregnant women aged between 18 and 45 years old up to 28 weeks of gestation, with singleton pregnancies and with no metabolic or cardiovascular pre-existing comorbidities (DM type 1 and 2 or chronic hypertension).

Exclusion criteria were: pregnant women aged less than 18 or more than 45 years old, women with pre-existing comorbidities (DM type 1 and 2 or chronic hypertension), multiple pregnancy, or any other comorbidity, ongoing medication treatment or disability that is significant enough to possibly impact on further course of mother or offspring wellbeing.

Gestational diabetes mellitus diagnosis was established according to the American Diabetes Association (ADA) recommendations for the 2 h 75 g oral glucose tolerance test (OGTT) as at least two values greater than a fasting glucose of 5.3 mmol/L, a 1 h glucose of 10 mmol/L, or a 2 h glucose of 8.6 mmol/L [20]. For moderate risk of GDM patients screening test was performed during the second trimester (between 24–28 weeks of pregnancy). For patients at higher risk (overweight or obese or having positive family history for DM, sibling or child with DM), screening was performed earlier in pregnancy on their first prenatal visit.

According to ADA recommendations all women who fulfilled study protocols were divided in two groups: pregnant women diagnosed with GDM and control group of patients who did not have GDM. The participants with confirmed GDM were prescribed combined diet and exercise intervention. Otherwise, if glucose levels were still not well regulated up to two weeks after diet and exercise were prescribed, oral anti-diabetic treatment (metformin) was introduced. None of the patients required insulin treatment.

### 2.2. Evaluated Parameters

Observed parameters were divided into four groups:

1. Maternal: Gestational Weight Gain (GWG), weight and body mass index (BMI) at delivery, comorbidities related to the pregnancy (pregnancy related hypertension (PRH), anemia in pregnancy, genitourinary infectious), drug use in pregnancy (antihypertensive drugs, antibiotics, and Low-weight-molecular heparins), presence of the congenital thrombophilia (CT), delivery mode, complication during delivery, prelabor premature rupture of membranes (PROM) and laboratory analysis results.
2. Fetal ultrasound parameters: amniotic fluid index (AFI), estimated fetal weight (EFW), head circumference (HC), biparietal diameter (BPD), femoral length (FL), abdominal circumference (AC), large for gestational age (LGA), and small for gestational age (SGA).
3. Neonatal: gender, gestational age, anthropometric values (birth weight, birth length, newborns head and chest circumferences), Apgar score, glucose and bilirubin levels, phototherapy requirements, hypotrophy/hypertrophy newborn and presence of birth injuries.
4. Infant motor development.

Pre-pregnancy anthropometric and personal medical data, including positive family history of diabetes type 1 or 2 were collected from primary health centers.

According to the World Health Organization (WHO) and the Institute of Medicine (IOM) recommendations, patients were divided into four different groups based on their Gestational Weight Gain (GWG) and preconceptional Body Mass Index (BMI). BMI bellow 18.5 kg/m^2^ was defined as underweight, BMI between 18.5–24.9 kg/m^2^ as normal weight, BMI between 25.0–29.9 kg/m^2^ as overweight and obesity was defined as BMI 30.0 kg/m^2^ and more. Gestational weight gain (GWG) was calculated as difference between maternal weight at delivery and before pregnancy. Based on IOM recommendations for singleton pregnancies excessive GWG cutoff value was 9.1 kg for obese, for overweight 11.5 kg, for normal weight 16 kg, and for underweight 18 kg [21,22].

Pregnancy related hypertension (PRH) is considered when values of blood pressure are above 140/90 mmHg after two separate measurements and after 20 weeks of gestation [23] and anemia in pregnancy was defined as hemoglobin concentration <110 g/L.

Oral antidiabetic drug use (Metforin), antibiotic use in pregnancy and during delivery, use of antihypertensive therapy and low-molecular-weight heparin (LMWH), positive urine culture (UC) and positive group B streptococcus (GBS) swab data were collected from patient’s medical record.

Four types of delivery mode were defined and assessed: vaginal, prostaglandins induced, Cesarean section, and instrumental. Complications during delivery were defined as postpartum hemorrhage (estimated blood loss more than 500 mL for vaginal delivery and more than 1000 mL for Caesarean section), uterine atony, retained placenta and postpartum manual exploration of the uterine cavity and instrumental revision of the uterine cavity.

The prelabor premature rupture of membranes (PROM) was defined as a rupture of fetal membranes before the onset of labor after completed 37 weeks of gestation [24].

The blood sample was collected twice for laboratory analysis, up to 24 h before delivery and 24 h after delivery. D dimer, glucose, hemoglobin, leucocytes, thrombocytes, as well as rhesus factor (positive or negative) were evaluated.

Ultrasonography parameters including: amniotic fluid index (AFI), biparietal diameter (BPD), head circumference (HC), abdominal circumference (AC), femoral length (FL), and estimated fetal weight (EFW) were evaluated up to three days before the delivery. Same sonographer has performed all ultrasonography measurements on the same ultrasound model (The Voluson™ E8 ultrasound system (GE Healthcare Dharmacon, Inc., Chicago, IL, USA).

Large for Gestational Age (LGA) was defined as newborns birth weight over 90th percentile for the appropriate gestational age and small for gestational age (SGA) was defined as newborns birth weight below the 10th percentile for the appropriate gestational age [25].

To calculate gestational age we have used Naegele’s rule and adjusted weeks into days. To verify the accuracy of esteemed gestational age according to Naegele’s rule, we used the first trimester ultrasonography to confirm the gestational age. Preterm birth was defined according to WHO recommendations as birth before completed 37 weeks of gestation.

Newborn’s glucose levels were measured in the first hour of life, and bilirubin levels were measured according to medical indications. Apgar score in the first and fifth minute, newborns weight and length were evaluated, and phototherapy requirement data was collected from newborns health data reports. Observed birth injuries in our study were brachial plexus injuries and cephalohematomas. Birth weight <10th percentile for gestational age was defined as hypotrophy, and birth weight >90th percentile for gestational age as hypertrophy. This data was collected from newborns medical history.

To assess infant’s early motoric development at 3 and 6 months of age, we used Alberta infant motor scale (AIMS). The AIMS test was performed by the trained resident under the supervision of board-certified specialist of physical medicine and rehabilitation. The AIMS is composed of 58 items. It is a non-referenced measure with high specificity and sensitivity for motor deficits. Out of 58 items, 21 are for pronation, 9 for supination, 12 for sitting and 16 for standing. At 3 months of infants age, pronation and supination were analyzed and at 6 months of age sitting and standing were additionally evaluated aside of pronation and supination [26].

### 2.3. Statistical Analysis

Continuous data were described with mean values (MV) and standard deviation (SD). Categorical data were described by numbers (*N*) and percents (%). Pearson chi-square test assessed differences between tested groups of categorical data. Koglomorov–Smirnov test was used to test normality of numeric data. Groups with normal distribution were compared by *t* test and for the rest of comparisons Mann–Whitney *U* test was applied. Univariate and multivariate regression models were used to test association of evaluated parameters.

It was estimated that the excessive GWG in pregnant women with GDM was significantly more frequent than in pregnant women without GDM (72% vs. 36.6%). Inclusion of 203 participants from this study, were frequency of those with GDM is *N* = 50, will accomplish 99.8% power to detect a significant difference in frequency of GWG in groups, at a two-tailed significance level of 0.05, using chi square test. Also, this simple size will accomplish 99,3% power to identify a significant difference in PRH. The *p* < 0.05 was considered as statistically significant. Statistical analysis was done by IBM SPSS Statistics for Windows, version 26.0 (IBM Corp., Armonk, NY, USA).

## 3. Results

Patients in GDM group had significantly higher preconceptional weight, preconceptional BMI, weight at delivery, BMI at delivery, and glucose values after delivery. They have also used antihypertensive drugs, antibiotics, and LMWH more frequently than the control group of patients. D dimer values after delivery (*p* = 0.033) were also higher in the GDM group (Table 1).

Fetal ultrasound parameters including HC (*p* = 0.006), BPD (*p* = 0.036), AC (*p* = 0.003), EFW (*p* = 0.009), LGA (*p* = 0.001) were statistically significantly higher in GDM group in comparison with the control group. Length at birth (*p* = 0.012), newborns head (*p* = 0.008) and chest (*p* = 0.018) circumference were also statistically significantly higher in GDM group as well (Table 2).

The values of all AIMS domains on both visits (3 and 6 months) were statistically significantly higher in the group of newborns from the mothers without diagnosed DM (for pronation three months (*p* = 0.008) and six months (*p* = 0.041), supination three months (*p* < 0.001) and six months (*p* = 0.007), sitting six months (*p* = 0.001) and standing (*p* = 0.022)) (Table 2).

**Figure 1:**
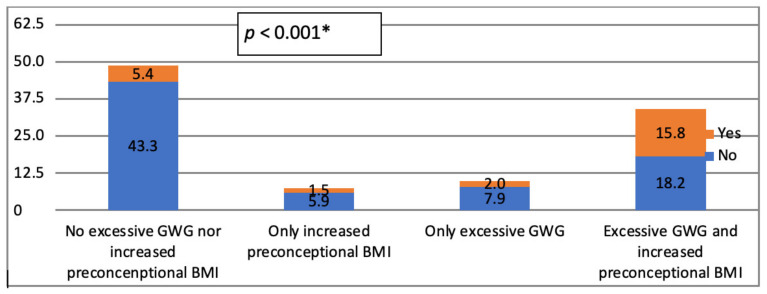
Frequencies of excessive GWG and increased pre-pregnancy BMI with regards to the presence of GDM in study group. * Chi-square test; GWG: Gestational Weight Gain; BMI: Body Mass Index; GDM: Gestational Diabetes Mellitus.

There is significant difference in distribution between two tested groups of patients regarding the presence of GWG and preconceptional BMI (*p* < 0.001) (Figure 1). Those without DM had most frequently no excessive GWG nor increased preconceptional BMI, while those with DM more frequently were with excessive GWG and increased preconceptional BMI.

The association of maternal, fetal ultrasound and neonatal parameters with GDM presence is presented in Table 3 by univariate logistic regression analysis.

Multivariate logistic regression analysis adjusted for maternal parameters regarding the presence of GDM was presented in Table 4 (Model 1). None of tested parameters were shown to be significantly associated (*p* > 0.05), except for positive family history for DM (*p* = 0.002) and mode of delivery (*p* = 0.021).

Multivariate logistic regression analysis adjusted for fetal and newborn perinatal parameters regarding the presence of GDM was presented in Table 5 (Model 2). None of tested parameters were shown to be significantly associated (*p* > 0.05), except for AFI (*p* = 0.012) and AIMS supination at three months of life (*p* < 0.001).

## 4. Discussion

Our findings demonstrated that positive family history for DM, obesity degree, PRH, and CT are among anamnestic data and maternal pregnancy-related complications with the strongest positive association with GDM. Positive family history for DM was 10 times more frequent in the group of patients diagnosed with GDM, and obesity degree was nearly three times more common. These results are in accordance with previously published data [27,28]. Even more, van Leeuwen and coauthors have proposed a model to estimate the probability of development of GDM from medical history and patient characteristics. Their prospective study pointed out that both family history and obesity degree could be used as clinical predictors for GDM development [29].

Our study has also showed that women diagnosed with GDM carried the burden of PRH three times more than women from control group. Obesity degree also had impact on the incidence of PRH and GDM, which is in line with other authors results [30,31].

Unlike the meta-analysis published by Sun et al. we have not found positive association between earlier menarche and increased risk for GDM [32]. Contrary to Kahn’s and coauthors work, we have found that CT is more frequent diagnosed in GDM group of women [33]. Those results could be explained with the fact that we do not perform genetic testing for CT unless in case of recurrent pregnancy loss. Nearly 90% of patients with GDM have previously performed genetic testing for CT unlike the control group in which less than 30% of participants underwent this type of testing. Furthermore, more than 85% of our patients were diagnosed with heterozygous mutations of methylenetetrahydrofolate reductase (C677T and A1298C) and plasminogen activator inhibitor-1 (4G/5G) mutations that did not require any specific anticoagulant therapy according to hematologist reports [34,35]. In Serbia, testing for CT is covered by the public health insurance.

Our study showed that all fetal biometric growth parameters were significantly higher in women with gestational diabetes than in women with normal blood glucose status, and these results are supported with the findings of other studies as well [36]. Both GDM and maternal pre-pregnancy obesity might have an individual as well as cumulative effects on fetal as well as on neonatal anthropometric parameters [37,38]. Additionally, in the study published by Chee et al. fetal AC was identified as a predictor of LGA. We have also found strong positive correlation between AC and GDM, leading us to believe that AC might be a potent parameter in early assessment of both GDM and LGA. Therefore, this parameter could be used also as a potential marker for the fetal monitoring and surveillance during pregnancy [37].

In our study, prevalence of LGA was 3.5 times higher in the study group, in comparison with the control group. Regarding these findings and given the strong positive association with the mode of delivery, it is expected that Cesarean section was preferable and optimal delivery mode in our study as well [39,40]. Participants in the study group had significantly higher pre-pregnancy and at delivery BMI as well as excessive GWG, and these contributing factors cannot be excluded in the overall analysis of delivery mode, since both BMI and GWG are associated with higher rates of Cesarean section [41,42,43]. Newborns weight as well as head circumference at birth were significantly higher in the GDM group of patients, unlike to newborns length and chest circumference, which is suggestive of cephalopelvic disproportion as more common indication for Cesarean section in the GDM group of patients. Rates of Cesarean section upon failed induction of labor were higher in the GDM group, five, compared to two patients in the study group. The fact that all five patients in the GDM group were overweight or obese makes the interpretation of the results challenging since it is well known that obesity is closely related with failed induction of labor [44]. Unlike to the other authors results [45], when stratified by age and parity, we have not found significant difference between two compared groups.

Amniotic fluid disorders resemble potential intrauterine jeopardy that fetus is exposed to, and GDM is the most common maternal risk factor that can cause amniotic fluid disorders [46]. Our study findings are in accordance with these findings, and we have found that excess of amniotic fluid index was significantly associated with GDM.

Even though all our patients have maintained good glicoregulation during the entire course of pregnancy, whether it was the due to diet managed GDM or the patients were treated with oral antidiabetic drugs (OAD), strong positive association was established between Apgar scores at first and fifth minute after birth and GDM. Other authors have established this association as well [47,48,49]. Postpartum glucose levels observed in our study oblige us to maintain further postpartum surveillance of GDM patients [50].

As it has been previous mentioned infants born to the mothers with GDM showed worse infant motors development results at the first and second visits (at three and six months of life). At the first visit the delay was just below five and a half times more frequent for supination on AIMS scale, versus pronation that was just above two times higher. The frequencies of delay according to the AIMS scale were different for selected motoric measurement at six months of life, where the highest frequency of more than three times was for standing and lowest (just below two times) for supination and sitting. The possible effects of gestational diabetes that was uncontrolled or poorly controlled on child motoric impairment were previously described in the study of Ghassabian et al. [51]. They stressed the possible teratogenic effect of hyperketonemia or complications that are related to pregnancy in gestational diabetes [51]. However, we should state that none of these parameters on both testing occasions were shown to be as significant predictors. The possible explanation could be the well-controlled GDM during the course of pregnancy.

Despite having very successful screening programs for GDM, modern therapeutic approaches, excellent monitoring tools for pregnant women and their babies GDM still represent a huge global health burden. Better understanding the association between GDM and maternal, fetal, and neonatal outcomes can help us find the most appropriate timing for diagnosis of GDM and for intervention. Early detection and prevention of all adverse events related to GDM is a core stone of modern approach to a GDM problem and its consequences [52].

Several limitations in this research should be addressed. Study individuals are from Serbian population thus specific variations and inherited disposition might be present in different populations. Another limitation to the study is physician’s knowledge of present GDM of mothers. Additional limitation refers to the study sample, therefore larger group of participants should be included to increase findings sensitivity.

## 5. Conclusions

Our findings demonstrate that numerous parameters are significantly associated with GDM, but only the AFI and AIMS supination at three months remained significantly associated in multivariate logistic regression model. Therefore, on time and proper screening of patients and adequate glicoregulation during the entire course of pregnancy lead to favorable perinatal outcome. However, despite the presence of significant association of numerous tested parameters in our study on perinatal outcomes and early motoric development, it is advisable to establish appropriate clinical decision—making strategies for on time inclusion and regular follow-ups of all pregnant women at risk. Ultimately, this will have positive impact on public health and overall quality of life of newborns and their mothers.

## Figures and Tables

**Table 1:** Maternal parameters characteristics according to GDM presence (*N* = 203).

Parameters		GDM		*p*
		Yes *N* = 50	No *N* = 153	
Age, years (MV ± SD)		32.74 ± 5.04	31.73 ± 4.73	0.200 *
Menarche (MV ± SD)		12.94 ± 0.98	13.08 ± 1.27	0.408 **
Rhesus type *N* (%)	Negative	7 (14%)	20 (13.1%)	0.867 ***
	Positive	43 (86%)	133 (86.9%)	
Allergies *N* (%)	Yes	7 (14%)	15 (9.8%)	0.407 ***
	No	43 (86%)	138 (90.2%)	
Positive family history for cardiovascular disease *N* (%)	Yes	17 (34%)	24 (15.7%)	0.005 ***
	No	33 (66%)	129 (84.3%)	
Positive family history for DM *N* (%)	Yes	13 (26%)	5 (3.3%)	<0.001 ***
	No	37 (74%)	148 (96.7%)	
Pre-pregnancy Weight (MV ± SD) (kg)		73.14 ± 12.16	65.61 ± 10.66	<0.001 **
Pre-pregnancy BMI (MV ± SD) (kg/m2)		25.31 ± 4.06	22.67 ± 3.40	<0.001 **
Obesity degree *N* (%)	Underweight	1 (2%)	4 (2.6%)	<0.001 ***
	Normal weight	14 (28%)	100 (65.4%)	
	Overweight	29 (58%)	42 (27.4%)	
	Obese	6 (12%)	7 (4.6%)	
Weight at delivery, kg (MV ± SD)		90.88 ± 12.94	80.96 ± 12.11	<0.001 **
Gestational weight gain (GWG) *N* (%)	Yes	36 (72%)	56 (36.6%)	<0.001 ***
	No	14 (28%)	97 (63.4%)	
BMI at delivery (MV ± SD) (kg/m2)		31.58 ± 4.38	27.97 ± 3.96	<0.001 **
Parity after delivery *N* (%)	1	28 (56%)	83 (54.2%)	0.825 ***
	2	18 (36%)	61 (39.9%)	
	3	3 (6%)	8 (5.2%)	
	4	1 (2%)	1 (0.7%)	
Pregnancy Related Hypertension (PRH) *N* (%)	Yes	8 (16%)	9 (5.9%)	0.025 ***
	No	42 (84%)	144 (94.1%)	
Congenital Thrombophilia (CT) *N* (%)	Yes	7 (14%)	6 (3.9%)	0.012 ***
	No	43 (86%)	147 (96.1%)	
Anemia in pregnancy *N* (%)	Yes	27 (54%)	62 (40.5%)	0.095 ***
	No	23 (46%)	91 (59.5%)	
Drug use in pregnancy *N* (%)	Yes	39 (78%)	86 (56.2%)	0.006 ***
	No	11 (22%)	67 (43.8%)	
Oral Antidiabetic drug use in pregnancy *N* (%)	Yes	17 (34%)	0 (0%)	<0.001 ***
	No	33 (66%)	153 (100%)	
Urine culture test *N* (%)	Positive	7 (14%)	14 (9.2%)	0.328 ***
	Negative	43 (86%)	139 (90.8%)	
Group B Strep swab test *N* (%)	Positive	6 (12%)	29 (19.0%)	0.258 ***
	Negative	44 (88%)	124 (81.0%)	
Antibiotics use during Labor *N* (%)	Yes	15 (30%)	47 (30.7%)	0.924 ***
	No	35 (70%)	106 (69.3%)	
Mode of delivery *N* (%)	Vaginal	19 (38%)	109 (71.2%)	<0.001 ***
	Cesarean Section	24 (48%)	31 (20.3%)	
	Instrumental	6 (12%)	7 (4.6%)	
	Induced	1 (2%)	6 (3.9%)	
Prelabor premature rupture of membranes (PROM) *N* (%)	Yes	5 (10%)	19 (12.4%)	0.677 ***
	No	45 (90%)	134 (87.6%)	
Complications during labor *N* (%)	Yes	7 (14%)	18 (11.8%)	0.676 ***
	No	43 (86%)	135 (88.2%)	
Hemoglobin level before (MV ± SD) (g/L)		117.96 ± 9.49	119.53 ± 9.73	0.323 *
Hematocrit before (MV ± SD) (L/L)		34.92 ± 2.38	35.19 ± 2.70	0.528 *
Platelets before (MV ± SD) (1 × 109/L)		217.90 ± 63.76	231.24 ± 57.49	0.106 **
Glucose level before (MV ± SD) (mmol/L)		4.74 ± 0.75	4.58 ± 0.65	0.163 **
D dimer before (MV ± SD) (mg/L)		1.83 ± 1.07	1.66 ± 0.96	0.107 **
Leucocytes after (MV ± SD) (1 × 109/L)		11.13 ± 2.84	10.90 ± 2.69	0.443 **
Hemoglobin level after (MV ± SD) (g/L)		102.39 ± 11.68	105.97 ± 11.68	0.04/1 **
Hematocrit after (MV ± SD) (L/L)		30.46 ± 3.45	30.71 ± 4.24	0.235 **
Platelets after (MV ± SD) (1 × 109/L)		201.43 ± 63.65	215.07 ± 56.67	0.085 **
Glucose level after (MV ± SD) (mmol/L)		5.66 ± 1.07	4.88 ± 0.80	<0.001 **
D-dimer after labor (MV ± SD) (mg/L)		1.60 ± 0.80	1.41 ± 0.81	0.033 **

* Independent samples *t*-test; ** Mann–Whitney *U*-test; *** Chi-square test; GDM: Gestational Diabetes Mellitus; MV ± SD: Mean Value ± Standard Deviation; DM: Diabetes Mellitus; BMI: Body Mass Index.

**Table 2:** Fetal and neonatal parameters characteristics due to the presence of gestational diabetes mellitus (*N* = 203).

Parameters		GDM		*p*
		Yes *N* = 50	No *N* = 153	
Days of gestation (MV ± SD)		275.92 ± 7.49	275.38 ± 8.972	0.715 *
Biparietal diameter (BPD) (MV ± SD) (mm)		95.72 ± 3.71	94.42 ± 3.54	0.036 *
Head circumference (HC) (MV ± SD) (mm)		347.10 ± 46.29	335.14 ± 13.41	0.006 *
Abdominal circumference (AC) (MV ± SD) (mm)		351.86 ± 23.66	342.13 ± 19.67	0.003 *
Femoral length (FL) (MV ± SD) (mm)		75.26 ± 2.90	73.93 ± 4.34	0.071 *
Estimated fetal weight (EFW) (MV ± SD) (mm)		3646.70 ± 512.43	3444.18 ± 421.86	0.009 *
Large for gestational age (LGA) (MV ± SD)		0.30 ± 0.463	0.10 ± 0.307	0.001 *
Amniotic fluid index (AFI) (MV ± SD) (mm)		139 ± 56.72	123.33 ± 28.46	0.247 *
Small for gestational age (SGA) *N* (%)	Yes	4 (8%)	10 (6.5%)	0.723 **
	No	46 (92%)	143 (93.5%)	
Sex (Gender) *N* (%)	Male	31 (62%)	82 (53.6%)	0.299 **
	Female	19 (38%)	71 (46.4%)	
Length at birth (MV ± SD) (cm)		53.58 ± 2.75	52.61 ± 2.17	0.012 *
Birth weight (MV ± SD) (g)		3675.40 ± 565.35	3453.33 ± 469.26	0.006 ***
Newborn’s head circumference (MV ± SD) (cm)		35.92 ± 1.52	35.41 ± 1.36	0.008 *
Newborn’s chest circumference (MV ± SD) (cm)		34.64 ± 2.07	34 ± 1.80	0.018 *
Apgar score first minute (MV ± SD)		8.34 ± 0.89	8.77 ± 0.60	<0.001 *
Apgar score fifth minute (MV ± SD)		9.50 ± 0.61	9.83 ± 0.44	<0.001 *
AIMS pronation 3 month (MV ± SD)		2.40 ± 0.60	2.66 ± 0.54	0.008 *
AIMS supination 3 month (MV ± SD)		2.34 ± 0.59	2.80 ± 0.42	<0.001 *
AIMS pronation 6 month (MV ± SD)		15.82 ± 0.39	15.92 ± 0.27	0.041 *
AIMS supination 6 month (MV ± SD)		8.62 ± 0.72	8.85 ± 0.47	0.007 *
AIMS sitting 6 month (MV ± SD)		6.48 ± 0.89	6.82 ± 0.54	0.001 *
AIMS standing 6 month (MV ± SD)		1.88 ± 0.33	1.99 ± 0.29	0.022 *
Glucose level newborn (MV ± SD) (mmol/L)		3.44 ± 0.77	3.80 ± 3.46	0.709 *
Bilirubin level newborn (MV ± SD) (µmol/L)		154.42 ± 55.81	173.37 ± 63.33	0.032 *
Phototherapy *N* (%)	Yes	7 (14%)	35 (22.9%)	0.179 **
	No	43 (86%)	118 (77.1%)	
Hypertrophic newborn *N* (%)	Yes	5 (10%)	5 (3.3%)	0.058 **
	No	45 (90%)	147 (96.7%)	
Hypotrophic newborn *N* (%)	Yes	2 (4%)	5 (3.3%)	0.818 **
	No	48 (96%)	146 (96.7%)	
Birth injuries *N* (%)	Yes	2 (4%)	2 (1.3%)	0.237 **
	No	48 (96%)	150 (98.7%)	
Preterm birth *N* (%)	Yes	3 (6%)	0 (0%)	0.322 **
	No	47 (94%)	151 (100%)	

* Mann–Whitney *U*-test; ** Chi-square test; *** Independent samples *t*-test; AIMS: Alberta Infant Motor Scale.

**Table 3:** Regression analysis of perinatal parameters characteristics due to the presence of gestational diabetes mellitus (*N* = 203).

Parameters	Univariate Logistic Regression Analysis Presence of GDM		
	Exp (B)	95% CI	*p*
Age	1.045	0.977–1.118	0.199
Pre-pregnancy weight (kg)	1.060	1.029–1.092	<0.001
Menarche	0.905	0.687–1.193	0.905
Rhesus type	1.083	0.428–2.735	0.867
Allergies	1.498	0.573–3.912	0.410
Positive family history for cardiovascular disease	2.769	1.335–5.743	0.006
Positive family history for DM	10.400	3.488–31.010	<0.001
Pre-pregnancy BMI (kg/m^2^)	1.209	1.105–1.323	<0.001
Obesity degree	2.939	1.758–4.912	<0.001
Weight at delivery (kg)	1.064	1.035–1.094	<0.001
Gestational weight gain (GWG) (kg)	4.454	2.213–8.965	<0.001
BMI at delivery (kg/m^2^)	1.220	1.124–1.325	<0.001
GWG and increased pre-pregnancy BMI	1.892	1.458–2.457	<0.001
GWG, increased pre-pregnancy BMI and increased BMI at delivery	5.574	2.807–11.066	<0.001
Parity	1.042	0.637–1.702	0.871
Pregnancy pelated hypertension (PRH)	3.048	1.107–8.388	0.031
Congenital thrombophilia (CT)	3.988	1.273–12.497	0.018
Anemia in pregnancy	1.723	0.906–3.278	0.097
Drug use in pregnancy	2.762	1.316–5.798	0.007
Positive urine culture test	1.616	0.613–4.262	0.332
Group B strep swab test	0.583	0.227–1.499	0.263
Antibiotics use during labor	0.967	0.482–1.938	0.924
Mode of delivery	1.765	1.193–2.611	0.004
Prelabor premature rupture of membranes (PROM)	0.801	0.283–2.273	0.677
Complications during labor	1.221	0.478–3.120	0.677
Days of gestation	1.008	0.970–1.046	0.699
Biparietal diameter (BPD) (mm)	1.118	1.011–1.237	0.029
Head circumference (HC) (mm)	1.032	1.006–1.058	0.014
Abdominal circumference (AC) (mm)	1.025	1.007–1.044	0.005
Femoral length (FL) (mm)	1.130	1.009–1.265	0.035
Estimated fetal weight (EFW) (g)	1.001	1.000–1.002	0.007
Large for gestational age (LGA)	3.670	1.655–8.136	0.001
Amniotic fluid index (AFI) (MM)	1.011	1.003–1.020	0.011
Small for gestational age (SGA)	1.243	0.372–4.154	0.723
Gender	0.708	0.368–1.361	0.300
Length at birth (cm)	1.210	1.042–1.405	0.012
Birth weight (g)	1.001	1.000–1.002	0.008
Newborn’s head circumference (cm)	1.310	1.031–1.666	0.027
Newborn’s chest circumference (cm)	1.219	1.010–1.471	0.039
Apgar score first minute	0.473	0.310–0.722	0.001
Apgar Score fifth minute	0.324	0.179–0.588	<0.001
Leucocytes before (1 × 10^9^/L)	0.984	0.853–1.136	0.830
Hemoglobin level before (g/L)	0.983	0.951–1.017	0.322
Hematocrit before (L/L)	0.961	0.849–1.087	0.526
Platelets before (1 × 10^9^/L)	0.996	0.990–1.002	0.171
Glucose level before (mmol/L)	1.418	0.891–2.255	0.141
D dimer before (mg/L)	1.179	0.870–1.598	0.289
Leucocytes after (1 × 10^9^/L)	1.032	0.918–1.160	0.598
Hemoglobin level after (g/L)	0.975	0.950–1.002	0.066
Hematocrit level after (L/L)	0.986	0.911–1.066	0.717
Platelets after (1 × 10^9^/L)	0.996	0.990–1.002	0.157
Glucose level after (mmol/L)	2.469	1.698–3.591	<0.001
D dimer after (mg/L)	1.315	0.905–1.909	0.151
AIMS pronation 3 month	0.468	0.253–0.864	0.015
AIMS supination 3 month	0.182	0.087–0.381	<0.001
AIMS pronation 6 month	0.388	0.153–0.984	0.046
AIMS supination 6 month	0.522	0.310–0.880	0.015
AIMS sitting 6 month	0.514	0.330–0.801	0.003
AIMS standing 6 month	0.304	0.107–0.866	0.026
Glucose level newborn (mmol/L)	0.935	0.763–1.146	0.515
Bilirubin level newborn (µmol/L)	0.995	0.989–1.000	0.063
Phototherapy	0.549	0.227–1.328	0.183
Hypertrophic newborn	3.267	0.905–11.793	0.071
Hypotrophic newborn	1.217	0.229–6.476	0.818
Birth injuries	3.125	0.429–22.786	0.261
Preterm birth	0.000	0.000	0.999

**Table 4:** Multivariate regression analysis of maternal parameters due to the presence of GDM (*N* = 203).

Parameters	Multivariate Logistic Regression Analysis Model 1 Presence of GDM		
	Exp (B)	95% CI	*p*
Pre-pregnancy Weight (kg)	0,987	0.873–1.117	0.840
Pre-pregnancy BMI (kg/m^2^)	0.874	0.603–1.266	0.477
Obesity degree	1.588	0.328–7.677	0.565
Weight at delivery (kg)	1.054	0.962–1.156	0.260
Gestational weight gain (GWG), increased pre-pregnancy BMI and increased BMI at delivery	2.727	0.794–9.361	0.111
Positive family history for cardiovascular disease	0.369	0.084–1.622	0.187
Positive family history for DM	20.088	3.049–132.324	0.002
Pregnancy related hypertension (PRH)	1.341	0.316–5.684	0.691
Congenital thrombophilia (CT)	3.304	0.752–14.520	0.114
Drug use in pregnancy	1.807	0.751–4.350	0.187
Mode of delivery	1.703	1.085–2.673	0.021

**Table 5:** Multivariate regression analysis of fetal and newborn perinatal parameters due to the presence of GDM (*N* = 203).

Parameters	Multivariate Logistic Regression Analysis Model 2 Presence of GDM		
	Exp (B)	95% CI	*p*
Biparietal diameter (BPD) (mm)	0.894	0.703–1.137	0.360
Head circumference (HC) (mm)	1.061	0.999–1.127	0.056
Abdominal circumference (AC) (mm)	1.032	0.963–1.106	0.378
Femoral length (FL) (mm)	1.247	0.970–1.604	0.086
Estimated fetal weight (EFW) (g)	0.996	0.991–1.001	0.140
Large for gestational age (LGA)	1.567	0.309–7.940	0.587
Amniotic fluid index (AFI) (mm)	1.018	1.004–1.033	0.012
Length at birth (cm)	1.041	0.674–1.606	0.857
Birth weight (g)	1.001	0.998–1.004	0.593
Newborn’s head circumference (cm)	1.124	0.627–2.017	0.694
Newborn’s chest circumference (cm)	1.021	0.566–1.841	0.945
Apgar score first minute	0.370	0.065–2.122	0.265
Apgar score fifth minute	0.726	0.064–8.238	0.796
AIMS pronation 3 month	0.923	0.343–2.487	0.874
AIMS supination 3 month	0.125	0.041–0.379	<0.001
AIMS pronation 6 month	0.389	0.033–4.535	0.452
AIMS supination 6 month	2.010	0.568–7.115	0.279
AIMS sitting 6 month	1.722	0.401–7.397	0.465
AIMS standing 6 month	0.798	0.118–5.396	0.817

## Data Availability

Original data are available on request.
